# Using systematic data categorisation to quantify the types of data collected in clinical trials: the DataCat project

**DOI:** 10.1186/s13063-020-04388-x

**Published:** 2020-06-16

**Authors:** Evelyn Crowley, Shaun Treweek, Katie Banister, Suzanne Breeman, Lynda Constable, Seonaidh Cotton, Anne Duncan, Adel El Feky, Heidi Gardner, Kirsteen Goodman, Doris Lanz, Alison McDonald, Emma Ogburn, Kath Starr, Natasha Stevens, Marie Valente, Gordon Fernie

**Affiliations:** 1 Health Research Board Clinical Research Facility, University of Cork, Cork, Ireland; 2 Health Services Research Unit, University of Aberdeen, Aberdeen, UK; 3 Centre for Healthcare Randomised Trials, Health Services Research Unit, University of Aberdeen, Aberdeen, UK; 4 Nursing, Midwifery and Allied Health Professions (NMAHP) Research Unit, Glasgow Caledonian University, Glasgow, UK; 5 Institute of Population Health Sciences, Queen Mary University of London, London, UK; 6 Primary Care Clinical Trials Unit, University of Oxford, Oxford, UK; 7 Pragmatic Clinical Trials Unit, Queen Mary University of London, London, UK; 8 Birmingham Clinical Trials Unit, University of Birmingham, Birmingham, UK

## Abstract

**Background:**

Data collection consumes a large proportion of clinical trial resources. Each data item requires time and effort for collection, processing and quality control procedures. In general, more data equals a heavier burden for trial staff and participants. It is also likely to increase costs. Knowing the types of data being collected, and in what proportion, will be helpful to ensure that limited trial resources and participant goodwill are used wisely.

**Aim:**

The aim of this study is to categorise the types of data collected across a broad range of trials and assess what proportion of collected data each category represents.

**Methods:**

We developed a standard operating procedure to categorise data into primary outcome, secondary outcome and 15 other categories. We categorised all variables collected on trial data collection forms from 18, mainly publicly funded, randomised superiority trials, including trials of an investigational medicinal product and complex interventions. Categorisation was done independently in pairs: one person having in-depth knowledge of the trial, the other independent of the trial. Disagreement was resolved through reference to the trial protocol and discussion, with the project team being consulted if necessary.

**Key results:**

Primary outcome data accounted for 5.0% (median)/11.2% (mean) of all data items collected. Secondary outcomes accounted for 39.9% (median)/42.5% (mean) of all data items. Non-outcome data such as participant identifiers and demographic data represented 32.4% (median)/36.5% (mean) of all data items collected.

**Conclusion:**

A small proportion of the data collected in our sample of 18 trials was related to the primary outcome. Secondary outcomes accounted for eight times the volume of data as the primary outcome. A substantial amount of data collection is not related to trial outcomes. Trialists should work to make sure that the data they collect are only those essential to support the health and treatment decisions of those whom the trial is designed to inform.

## Introduction

Data collection consumes a large proportion of trial resources. Each data item requires time and effort for collection, processing and quality control procedures [1]. Generally speaking, more data equals a heavier burden for trial staff and participants. More data may also increase the cost of the trial.

Outcomes are also not created equal. Trials usually have one outcome that is considered primary—the outcome of highest importance in the trial. Ideally, this outcome should be of most interest to stakeholders such as patients, clinicians, policymakers and funders. The primary outcome is normally the outcome used in the sample size calculation [2]. However, it is rare for a single outcome to provide enough information about the trial intervention(s) on its own, so it is usual to have secondary outcomes to provide a wider context for the main research question. These outcomes will be detailed in the trial protocol but tend not to drive sample size because they are rarely included in sample size calculations.

An under-recognised challenge in conducting a clinical trial is ensuring that the data collected are sufficient to answer the trial research questions but are not so substantial as to threaten the feasibility of the trial. As the primary outcome is the measurement that will be used to evaluate the effectiveness of the intervention, it seems reasonable to expect that it will receive a substantial part of the data collection effort (and funding). It is not clear that this is currently the case.

Data collection covers more than outcomes. Trials collect demographic, medical history, safety and regulatory non-outcome data, as well as generating their own data in the form of identifiers and process checks (e.g. ‘was leaflet X handed out? Y/N’). For drug trials in particular, evaluations may be focused on differentiating similar, competing therapies by incremental differences in one or all of safety, efficacy [3] and health economic measures, which can require a substantial amount of data. Growing requirements and requests from regulators and sponsor organisations have also increased the amount of data that routinely needs to be collected in clinical trials [3–5]. The amount of secondary, exploratory and auxiliary data in trials can be substantial, with more than half of the outcome data collected for Phase II and Phase III trials being less essential or supplementary [3]. One study of six drug/nutritional supplement trials found 137,008 items of data for 126 participants, of which the authors considered only 18,124 to be key for targeted risk-based monitoring purposes, or just 13% of all data collected [6].

Trials are collecting more data than ever. Getz and colleagues [7] examined 9737 trial protocols of Phase II and Phase III trials and found that between two time periods (2000–2005 and 2011–2015) the number of planned study visits had increased by 23% (Phase II) and 25% (Phase III). Similarly, the total number of trial procedures had also increased by 67% and 70%, respectively. Both increased data collection requirements. Apart from extra workload, these increases can also adversely affect recruitment and retention rates [7–9]. ‘How can trials be designed to minimise burden on staff and participants and how does this affect retention?’ was ranked third in a recent prioritisation exercise of unanswered research questions on trial retention [10]. Collecting large amounts of data may also increase missing data. This will reduce the power of the study if it affects the primary outcome and will reduce the power of already potentially underpowered secondary outcome analyses, especially if the data arenot missing at random [11]. Added to this is the possibility that after doing a lot of work, the data are not used anyway. O’Leary et al. [12] found that large proportions of collected data (median 82%, range 73–89%) were not reported in associated publications.

We wanted to examine the type and volume of data being collected across a variety of clinical trial types. This paper describes work done in the DataCat project in which we (1) developed a list of categories of data collected in trials and (2) applied that categorisation system to the data collected in trials run in the UK and Ireland. The work was unfunded and done as part of the Trial Forge initiative (www.trialforge.org) to improve trial efficiency.

## Methods

The original idea for this project came from a workshop held at the annual meeting of the UK Trial Managers’ Network (UKTMN) in London, UK, on 10th September 2015. ST facilitated the workshop, and around 20 trial managers suggested, then ranked, ideas for research projects addressing important questions relevant to them. The question ‘Why do we collect the data we do: what is the purpose of the data we collect on the CRF (case report form) and do we actually use it?’ was ranked second and formed the basis of the work done here. (The top ranked question, about electronic versus paper data collection, was considered more difficult to study without funding.) The work described here addresses the first half of the question, i.e. ‘What is the purpose of the data we collect on the CRF?’

Shortly after the meeting, we formed a group, mainly composed of trial managers but also trial methodologists, interested in addressing this question; many had attended the London workshop. Over time we expanded the group slightly to include people not present at the workshop, including the addition of a trial data manager.

The work had three stages:

- We developed a standard operating procedure for categorising data.
- We piloted the categories.
- We applied the tested categorisation list to a larger sample of trials.

### Stage I: development of a standard operating procedure for data categorisation

We developed the list of categories iteratively through a series of teleconference discussions where potential categories of data were considered. We drew the data categories from general features of trial protocols (e.g. demographics, primary outcomes, secondary outcomes), our experience of running trials (e.g. data required to identify participants or link data) and our experience of initial attempts to apply the list to trial data collection forms (case report forms [CRFs] and participant questionnaires) from three trials (AMBER [13], KAT [14] and SALVO [15]). We identified these trials primarily from personal involvement with the trial; none was a Clinical Trial of an Investigational Medicinal Product (CTIMP). We were interested in definitive trials, often called Phase III trials. Based on this, a standard operating procedure (SOP) was developed for how to categorise the data (see Additional file 1).

### Stage II: piloting of the categories

We piloted the categorisation list and SOP with six trials, including the three with which we developed the process. The three additional trials were also selected based on personal involvement in the trial; we quickly found in Stage I that categorisation needed someone close to the trial. We first listed all the data items collected on the trial data collection forms. We defined data items as the individual pieces of data collected on the trial data collection forms (also called fields or variables). We then assigned each data item on the list to a single data category that best described what type of data it was (i.e. the main reason it was collected). We used the trial protocol to help with this process. Where data items were dependent on events occurring (e.g. repeat visits, an adverse event, drug prescription), the number of data items categorised did not necessarily represent the maximum possible number. Two team members independently categorised the data for each trial, one who was familiar with the trial and one who was not. The two reviewers then met or had a telephone call to discuss and resolve any discrepancies in their categorisations. Some data items were hard to categorise, and discrepancies often involved one of the two reviewers using ‘Miscellaneous’ where the other did not. For example, in SALVO [15] ‘Mother ABO blood group’ was initially categorised as ‘Miscellaneous’ by one person and ‘Medical history’ by the other. After discussion the two team members agreed that in this case this data item was best described as ‘Miscellaneous’. If necessary, the rest of the project team was consulted to resolve a discrepancy.

Having piloted the categorisation list, a new member of the project team (EC) reviewed the same six trials and all sources of discrepancy together with one member of the project team (ST). We refined the categories list and guidance where necessary with agreement from the whole group.

### Stage III: application of the tested categorisation list to a larger sample of trials

We then categorised data items collected in an additional sample of 12 trials (also a convenience sample) using the categorisation list and SOP. As with the pilot, we identified the trials primarily through the personal knowledge of the trial by a project team member. In this final stage we included CTIMPs along with additional non-CTIMPs.

## Results

### Stages I and II (creating the categorisation list, standard operating procedure and piloting)

The final list of categories included 17 types of data (Table 1) together with guidance on the type of data each category might contain. This included a hierarchical categorisation process which was developed to handle situations where a data item could potentially be placed into more than one category (see Additional file 1 for the guidance document we used when making category decisions).

**Table 1:** The 17 data collection categories

Category	Example
Outcomes	
1. Primary Outcome	As identified in the trial protocol
2. Primary outcome but not primary analysis	Primary outcome is weight loss at 12 months but weight loss is also measured at 3 months
3. Secondary Outcomes	As designated in the trial protocol
4. Outcome data not listed as primary, secondary or health economics outcome	
5. Items from a core outcome set	
6. Health Economics	
Non-outcomes	
7. Participant identification items	Participant ID, postal contact details, general practitioner name and contact details
8. Items needed for randomisation	Age, sex, site
9. Eligibility	
10. Demographics	Age, sex, family history of condition of interest
11. Medical History	
12. Data Management Item	Visit number
13. Safety Data	Concomittant medications
14. Regulatory Data	Deviation logging, reason for withdrawal
15. Compliance Data	Dose administered, tablets taken or returned, confirmation of completed processes, randomisation allocation
16. Process Outcomes	How much blood was collected, who delivered the educational information
17. Miscellaneous	

The review of the six pilot trials led by EC resulted in one new category (‘Primary outcome but not primary analysis’) and some additional guidance to reduce ambiguity around category choice. The extra category was added to account for cases where the primary outcome (e.g. weight) was measured but at a timepoint (e.g. 12 weeks) that was not the primary outcome measurement point (e.g. 12 months). The SOP was also updated (see Additional file 1).

#### 3.2. Stage III

A total of 18 trials were categorised, including the six used in the pilot; 15 complex intervention non-CTIMPs and three CTIMPs, predominantly publicly funded and sponsored by UK academic and/or National Health Service (NHS) institutions. All trials were superiority trials. The 18 trials had different chief investigators, and the teams were either completely, or largely, different. There was some overlap of individuals (e.g. ST was involved in ActWELL and ECLS; EAGLE and TAGS involved some of the same clinicians), but the majority of the trial team were different across all 18 trials. The oldest began in 1998 with the most recent beginning in 2017. The characteristics of the included trials are presented in Table 2. Duration of follow-up ranged from a few days (until discharge) to 10–15 years, and target sample sizes ranged from 40 to 12,000 trial participants. Trial data collection documents were received from five different institutions in the UK and Ireland.

**Table 2:** Characteristics of included categorised trials

Acronym	Full title	Funder	Funding start – end (month/year)	Follow-up duration	Sample size	Trial received from
ActWELL	A randomised controlled trial to assess the impact of a lifestyle intervention (ActWELL) in women invited to NHS breast screening	The Scottish Government	Jan 2017 – Dec 2019	12 months	414	University of Dundee
AMBER	Abdominal massage for neurogenic bowel dysfunction in people with multiple sclerosis	National Institute for Health Research - Health Technology Assessment (NIHR-HTA) programme	July 2014 – June 2017	6 months	200	NMAHP Research Unit, Glasgow Caledonian University
CONFIDeNT	CONtrol of Faecal Incontinence using Distal NeuromodulaTion	NIHR-HTA	Feb 2012 – Apr 2014	14 weeks	212	Queen Mary University London
EAGLE	Whether removal of the lens of the eye (lens extraction) for newly diagnosed primary angle closure glaucoma results in better patient-reported health vision, lower intraocular pressure and other outcomes compared with standard management	NIHR - Efficacy and Mechanism Evaluation (NIHR-EME)	Nov 2008 – July 2015	3 years	419	CHaRT, University of Aberdeen
ECLS	Detection in blood of autoantibodies to tumour antigens as a case-finding method in lung cancer using the EarlyCDT-Lung test	Chief Scientist Office, Scottish Government and Oncimmune Ltd	Aug 2013 – Aug 2018	24 months	12,000	University of Dundee
EMPiRE	AntiEpileptic drug Monitoring in PREgnancy: an evaluation of effectiveness, cost-effectiveness and acceptability of monitoring strategies	NIHR-HTA	Sept 2011 – Apr 2016	21 weeks approx.	1000	Queen Mary University London
Acronym	Full title	Funder	Funding start – end (month/year)	Follow-up duration	Sample size	Trial received from
HEALTH	A multicentre randomised controlled trial comparing laparoscopic supra-cervical hysterectomy with second generation endometrial ablation for the treatment of heavy menstrual bleeding	NIHR-HTA	Jan 2014 – Sep 2018	15 months	648	CHaRT, University of Aberdeen
HIP	Management of Hypotension In the Preterm: a multicentre, randomised controlled trial of hypotension management in the extremely low gestational age newborn	European Commission within the 7th Framework Programme	Oct 2010 – Sep 2017	2 years	340	INFANT Centre, Cork, Ireland
iQuaD	A multicentre randomised controlled trial comparing oral hygiene advice and periodontal instrumentation for the prevention and management of periodontal disease in dentate adults attending dental primary care	NIHR-HTA	Apr 2011 – Dec 2016	36 months	1860	University of Dundee & CHaRT, University of Aberdeen
KANECT^a^	The use of ketamine as an anaesthetic during electroconvulsive therapy (ECT) for depression: does it improve treatment outcome?	Chief Scientist Office - Scottish Government Health Directorate	Apr 2011 – Apr 2014	1 month	40	CHaRT, University of Aberdeen
KAT	Knee Arthroplasty Trial	NIHR-HTA	Dec 1998 – June 2023	10–15 years	2450	CHaRT, University of Aberdeen
PIMS	A randomised controlled trial comparing face-down and face-forward positioning after eye surgery for macular holes to see if this improves the rate of macular hole closure	NIHR-HTA	Apr 2015 – Apr 2018	3 months	192	Queen Mary University London
SALVO	A Randomised Controlled Trial of Intra-Operative Cell Salvage during Caesarean Section in Women at Risk of Haemorrhage	NIHR-HTA	Oct 2012 – Oct 2016	Until discharge	3050	Queen Mary University London
Acronym	Full Title	Funder	Funding start – end (Month/Year)	Follow-up Duration	Sample Size	Trial Received From
SUSPEND^a^	Use of drug therapy in the management of symptomatic ureteric stones in hospitalised adults: a multicentre placebo controlled randomised trial of a calcium channel blocker (nifedipine) and an α-blocker (tamsulosin)	NIHR-HTA	Jun 2010 – Oct 2014	12 weeks	1200	CHaRT, University of Aberdeen
TAGS	Treatment of Advanced Glaucoma Study (TAGS): a multicentre randomised controlled trial comparing primary medical treatment with primary trabeculectomy for people with newly diagnosed advanced glaucoma	NIHR-HTA	Jan 2014 – Jan 2020 ^a^(LTFU - Dec 2023 not included)	2 years	440	CHaRT, University of Aberdeen
TWICS^a^	A randomised, double-blind placebo controlled trial of the effectiveness of low dose oral theophylline as an adjunct to inhaled corticosteroids in preventing exacerbations of chronic obstructive pulmonary disease	NIHR-HTA	July 2013 – Dec 2017	12 months	1424	CHaRT, University of Aberdeen
ViDiFlu	Cluster-randomised, double-blind, placebo-controlled trial of vitamin D supplementation for the prevention of influenza and other respiratory infections	NIHR - Programme Grants for Applied Research	Mar 2010 – Apr 2013 (LTP)	12 months	108 units, - approx. 3 participants per unit	Queen Mary University London
VUE	Two parallel randomised controlled trials of surgical options for upper compartment (vault or uterine) pelvic organ prolapse	NIHR-HTA	Nov 2012 – Jan 2018	12 months	800	CHaRT, University of Aberdeen

^a^CTIMP

Proportions of data items within each trial were calculated and those proportions summarised across all 18 trials. Table 3 presents these categorisation results. Primary outcome data accounted for 5.0% (median)/11.2% (mean) of all data items collected. Secondary outcomes constituted the largest proportion of data items collected per participant per trial summarised across all included trials (median 39.9%; mean 42.5%). Two trials are notable exceptions to this trend (see Table 2): in ViDiFlu (a National Institute for Health Research [NIHR] programme grant-funded trial of vitamin D for prevention of influenza and other respiratory infections), primary outcomes accounted for the largest proportion of data items collected (50.3%), while in HIP (an EU-funded trial of hypotension management in extremely low gestational age newborns), safety data (21.1%) and compliance data (21.1%) accounted for the largest proportions of data items collected. Indeed, in ViDiFlu so many data items were for primary outcomes (5096 or 78.1% of the overall total from all 18 trials) that their inclusion inflates the average proportion of primary outcomes. These exceptions may relate to the nature of the trials—for example, HIP involves newborns, which may explain why it collected a lot of safety and compliance data—or to the trials’ funding streams, which were different to the majority of trials in our sample, which were largely funded by NIHR-Health Technology Assessment (HTA) or the Chief Scientist Office of the Scottish Government.

**Table 3:**
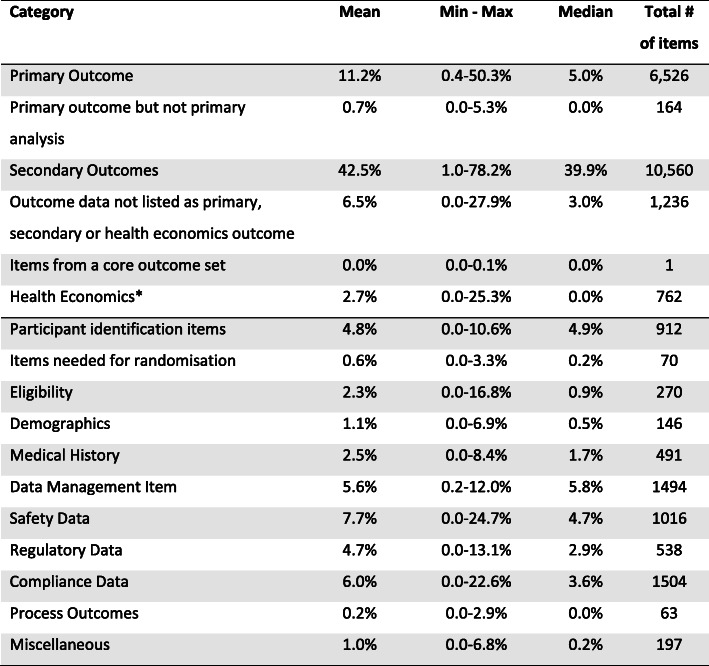
Mean, min-max and median proportion values and total number of items for all 17 data categories across all trials

Proportions of data items within each trial were calculated and those proportions summarised across all 18 trials. Items above the dividing line are considered outcome data, those under the line non-outcome data

^a^The Health economics category was only used when these outcomes were not explicitly listed as a primary or secondary outcome

Table 4 summarises the categorisation results across CTIMPs and non-CTIMPs separately. For CTIMPs, secondary outcomes (median 33.8%; mean 24.8%), safety data (16.9%; 16.4%), regulatory data (11.5%; 11.7%) and participant identification items (9.9%; 8.9%) constituted the largest proportion of data items collected.

**Table 4:**
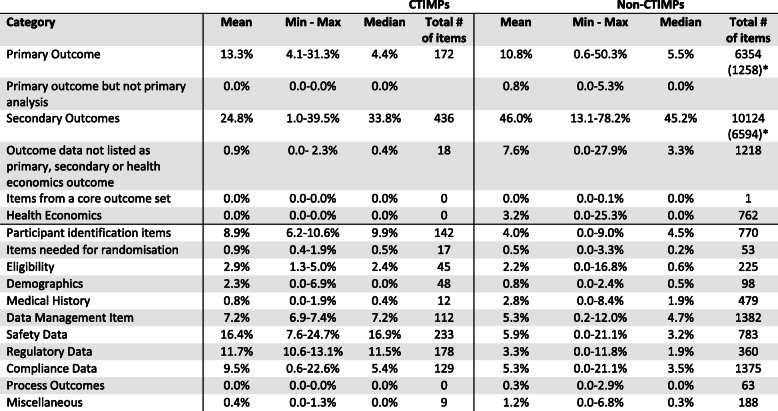
Mean, min-max and median proportion values and total number of items for all categories across CTIMPs and non-CTIMPs

^a^Totals without ViDiFlu data

For non-CTIMPs, secondary outcomes (median 45.2%; mean 46.0%), primary outcomes (5.5%; 10.8%), data management outcomes (4.7%; 5.3%) and participant identification items (4.5%; 4.0%) constituted the largest amount of data items collected.

Non-outcome data accounted for the vast majority (median 61.7%; mean 61.1%) of data items collected in CTIMPs as compared with about a third (27.4%; 31.6%) of data items collected in non-CTIMPs.

## Discussion

### General results

For our 18 trials a small proportion of all the data items collected, a median of 5.0%, were for the primary outcome assessment—the outcome assessment considered by the trialists themselves to be of the highest importance in their trial. The median of 5.0% is the better measure due to one trial, ViDiFlu, devoting half of its data items to the primary outcome.

Clearly the primary outcome is not the only thing that is important. One outcome is unlikely to provide all the information needed to make a judgement about the treatment being tested, meaning secondary outcomes are necessary. We also need to know something about harms, as well as cost. Participant identifiers and some data management and process information will also always be needed. Trials may also need to measure more than a single primary outcome.

In our sample the median proportion of secondary outcome data items is eight times that of the primary outcome. The ratio of non-outcome data to primary outcome data was similar. This might be fine. However, the undeniable design importance of the primary outcome, together with its importance to external judgements about the utility of the intervention, makes this distribution of attention look odd. At a minimum it is worthy of some reflection.

Our study raises three key questions:

1. Given that the primary outcome is the most important and (likely) the only fully powered outcome, is the substantially larger proportion of data collected for secondary outcomes justified?
2. Do people really appreciate how much non-outcome data trials collect?
3. Does volume of data correlate with data collection effort?

Our study answers none of these questions. However, it does highlight how important it is to try to answer them. Data collection itself is hard work, and it generates additional work by requiring data management systems, quality assurance and, usually, data entry to deal with it. Given the undoubted importance of the primary outcome, we need to be sure that all outcomes in our set of secondary outcomes—many, if not all, underpowered—are worth the effort. If data collection effort does relate to data volume, then it seems disproportionate for trial teams to devote around eight times as much effort on the secondary outcomes as on the primary. Secondary outcomes may support understanding of the primary outcome result, but they are not the outcomes that trialists themselves consider to be of most importance. A new Trial Forge project called ORINOCO (Optimising Resource Use in Outcome Collection) will look at data collection effort by collecting time spent collecting primary and secondary outcomes (https://www.abdn.ac.uk/hsru/what-we-do/research/projects/orinoco-826.php).

Why trial teams collect so much data is unclear but, anecdotally, we (and others) know that some investigators believe that since the participant is providing data anyway, why not collect a few more things? The work involved in doing this is unlikely to be borne by the person making the request. Items unrelated to the original aims of the trial are added, and trial team members have their own interests, and each adds something to the data collection job. Additional items can be added by Trial Steering Groups and, for that matter, funders. The tendency always seems to be upwards when it comes to data collection.

That said, our own anecdotal experience, and that of others [16], is that when the going gets tough with outcome collection, trial teams quickly start to focus on getting just primary outcome data from participants. This brings into stark focus the relative importance of those secondary outcomes. Secondary outcomes can address related questions and provide context in which to interpret the primary outcome, but we need to keep their relative importance in mind when selecting how many of them to collect. For definitive Phase III trials such as those we selected, a secondary outcome, like the primary, should be essential to the people (generally patients, healthcare professionals and policymakers) whose decisions the trial is intended to support. Anything else is garnish, which has clear resource implications in the cash-limited world of publicly funded research.

The amount of non-outcome data was a surprise. That a median of just under 5% of all data collected is linked to the participant ID was not a result we expected, nor was the finding that internal data management items (e.g. ticking a box if a process was completed) was almost 6%. Some of this cannot be avoided, but even here there is likely to be scope for efficiencies. For example, the proportion of data items linked to demographics ranged from 0 to 6.9%, with a median of 0.5%. Across most of our trials, around 2% of data were demographic. Trial designers should ask themselves at the beginning of trials what a reasonable volume of demographic (or other) data is, make sure they are resourced to collect at that level and have a clear use for these data once collected.

Our data underline that non-outcome data represent a substantial proportion of the data that participants need to provide and trial staff need to work with. Reducing the burden of trials on participants and staff was highlighted as an area in need of research to improve retention by the PRIORITY-2 project [10], and ways of assessing burden have been proposed [17]. One must also carefully choose the non-outcome data that will be collected so trial budgets and resource can be allocated proportionately. Those making policy and governance decisions about research (e.g. sponsors, regulators) need to weigh up their requirements for non-outcome data against the work needed to collect and manage it. Although we only included three CTIMPs, the impact of regulation (or at least how regulation is interpreted) on data collection workload is visible: for our three CTIMPS a median of 11.5% of all data items collected were classed as regulatory, compared to 1.9% for non-CTIMPs. Regulatory decisions are capable of directly leading to thousands of extra items of data collection across hundreds of trials. Regulators need to be confident that, on balance, their requirements do more good than harm and increase the transparency of their requirements. Grey areas around what is needed to meet the conditions imposed can lead to over-collection of data, as researchers may not be clear about what exactly is required. Some of the potential harm is workload, particularly if conservative interpretation, or misinterpretation, of legislation by research administrators adds additional but unnecessary data collection requirements [18].

### Limitations and strengths

Our work has limitations. The 18 included trials were a convenience sample rather than a random sample of published trials. We quickly found that the categorisation process required someone close to each included trial, and choosing trials that none of the team knew of made categorisation difficult. As such, we do not claim that our results are representative of all trials. However, all the included trials are real trials, not hypothetical ones, and they vary enormously in terms of trial teams, intervention, sample size and follow-up durations. We would be surprised if the headline result of substantially more data items dedicated to secondary outcomes than primary outcomes was overturned in a bigger sample. Our categorisation method (see Additional file 1) can be replicated by others for their own trials, and we could perhaps build up a larger sample over time.

Only three CTIMPs were included in our sample, which limits what we can say about a comparison between CTIMPs and non-CTIMPs. There appear to be more regulatory and safety data collected in CTIMPs, but to determine exactly how much requires rather more CTIMP trials. Moreover, some regulators (e.g. the UK’s Medicines and Healthcare products Regulatory Agency, the MHRA) categorise CTIMPs by risk, which means that not only would we need a larger sample, but also a good mix of CTIMP risk categories.

The categories were not always exclusive, and much of the discrepancy discussion between the reviewers categorising each trial amounted to which category won out given that a case could be made for more than one. Our SOP and guidance provided some rules. Generally, we went with the emphasis given in the trial protocol for outcomes and tried to be consistent for non-outcome data. Different pairs of reviewers may have reached slightly different conclusions for some items of data, although we are confident that the process we used was as robust as it could be for these judgement-based decisions.

There are some strengths too. The study idea came from trial managers and addressed a question that was very important to their trial front-line experience: what sorts of data are collected in trials? We are confident of the importance of the topic covered by this work. The study also involved staff with diverse roles from seven trials units in three regions with differing regulatory environments (England, Ireland and Scotland), which brought a range of perspectives. We have created a set of data categories, a SOP, guidance and some templates (see Table 1 and Additional file 1) that others can now use to assess their own trials, including at the design stage. Finally, the project started some new collaborations and has some simple messages that we think are worth all trialists’ attention.

### Implications for practice

1. For Phase III trials, we think trialists should continue to consult with patients, health professionals and policymakers to identify the outcomes they will need to inform their future decisions about the usefulness of the intervention being tested. Trialists should then resist requests to add to this list without having a compelling reason for collecting data not essential to stakeholders’ future treatment decisions. Core outcome sets [19] may help.
2. Trial teams could consider categorising the data they propose collecting on their CRFs before they start to use them. They could then check that the distribution of data volume is what they anticipated and want. This information would support decision-making for resource allocation to collect, process and quality control the data.

### Implications for future research

1. Measuring data collection effort. The time actually spent collecting data items is the focus of a new Trial Forge data collection project: ORINOCO will examine the time spent collecting primary and secondary outcomes (https://www.abdn.ac.uk/hsru/what-we-do/research/projects/orinoco-826.php).
2. Expansion of this work to assess a larger number of trials potentially with a focus on CTIMPs, given the small sample included here, would be beneficial.
3. The work described here did not assess whether collected data were actually used, or published. Doing so would be a useful addendum to this work, perhaps along the lines of the study done by O’Leary et al. [11].
4. The impact on data volume and distribution of doing one or both items listed under ‘Implications for practice’ would be worth evaluating. Like anything else, they are only worth doing if they lead to benefit. We would anticipate that they will reduce both volume of data and number of outcomes, especially secondary outcomes, but this needs to be demonstrated.

## Conclusion

Our results show that a small proportion of data collected in the studied trials was related to the primary outcome, while a substantial amount was not related to trial outcomes. Generally speaking, secondary outcomes account for eight times the volume of data as the primary outcome.

The data collection load is driven by the trial protocol. It is important that those designing trials ensure that the protocol focuses efforts on collecting data essential to support the health and treatment decisions of those whom the trial is designed to help. Collecting additional data threatens the key purpose of the trial and may be considered wasteful in the context of limited public funding for clinical research.

## Supplementary information

**Additional file 1.** Standard operating procedure for categorising data collected in trials

## Data Availability

Not applicable.

## Abbreviations

CRF: Case report form
CTIMP: Clinical Trial of an Investigational Medicinal Product
ID: Identifier
SOP: Standard operating procedure
UKTMN: UK Trial Managers’ Network
