# Supplementary material for: Using systematic data categorisation to quantify the types of data collected in clinical trials: the DataCat project
Source: Trials. 2020 Jun 16;21:535. doi: 10.1186/s13063-020-04388-x (PMC7298750; doi:10.1186/s13063-020-04388-x)
Supplement: Supplementary file 1 — Additional file 1. Standard operating procedure for categorising data collected in trials [file 13063_2020_4388_MOESM1_ESM.pdf]

TRIAL FORGE

Standard operating procedure for categorising data collected in trials

Version: 2.0
Date: 10/01/2018
Author(s): Shaun Treweek, University of Aberdeen;
Evelyn Crowley, HRB Clinical Research Facility, Cork

1. Purpose

This document describes the procedure for extracting and then categorising data items collected in trials.

2. Procedure

2.1. Data Categories

2.1.1. This SOP should be used together with the list of data categories contained in the Google Sheets document at https://docs.google.com/spreadsheets/d/16jJq25M3pD79tjLEFBKwQxJjGCsX-0UKSJD2fE5bFk8/edit#gid=0

2.2 People doing data extraction and categorisation

2.2.1 Extraction and categorisation of the data collected in each trial should be done by two people: one person should be closely connected to the trial, the other should be independent of the trial.

2.2.2 The person who is close to the trial should send a copy of the trial protocol to the person independent of the trial.

2.2.3 Each person should start from the same set of data collection forms. The actual data collection forms used by people collecting trial data should be used. Generally these forms will be on paper but electronic versions are fine so long as they are the actual data collection forms used in the trial rather than a database used only by IT staff. Whichever version is selected should be then used by both individuals doing categorisation.

2.2.4 Before starting categorisation, the two individuals should agree the list of data items contained in the data collection forms and create a single electronic data categorisation form listing these data items, which they will both use. All the fields to be categorised should be listed on this form. Examples of these forms are available from Shaun Treweek (streweek@mac.com).

2.2.5 If the two people cannot agree on a categorisation, they should discuss it with the whole project team and the team will reach a consensus view.

2.3 Specific rules for categorisation

2.3.1. All scheduled trial measurement points (e.g. baseline, 1-month, 6-months) will be considered.

2.3.2. Some data items could be placed in more than one data category. The guidance given in the Google Sheets document provides a hierarchy of categorisation. The key rule is to place the item into the category for which the data were primarily being collected.

2.3.3. For checkboxes headed 'Tick all that apply' (or similar) we will categorise all check box options. In other words if there were 10 options, we will categorise 10 fields.

2.3.4. For checkboxes headed 'Tick only one' (or similar) we will categorise only the overall heading for the data collection question not each individual field. In other words, we will categorise 1 field.

2.3.5. Any details collected to help with identification of the participant (including next best contact person etc.) should be categorised as 'Participant identification'.

2.3.6. Patient diaries will be included if they form part of the main data collection. All items within the diary should be categorised.

2.3.7. Where a data collection item is dependent on an event or on a previous response to a question, this should be recorded on the categorisation form as 'Dependent'. This may also apply to whole data collection forms e.g. the serious adverse event form. If possible, the person closely connected to the trial should estimate the proportion of participants for whom these items are completed.

2.3.8. All data items not marked 'Dependent' will be considered to be mandatory, although this need not be recorded explicitly on the data categorisation form.

2.3.9. Where an outcome is not explicitly listed as primary or secondary in the trial protocol, this data item will be listed in "Outcome Data not listed as primary, secondary or health economics outcomes".

2.3.10. Where a health economics outcome is listed as a secondary outcome in the trial protocol, this will be categorised in "Secondary outcomes".

2.3.11. Where a process outcome is listed as a secondary outcome in the trial protocol, this will be categorised in "Secondary Outcomes".

2.3.12. Where a process outcome is not listed as a secondary outcome in the trial protocol, this will be categorised in "Process outcomes".

2.3.13. Where data on the primary outcome is collected prior to the specified primary outcome measurement time point, this will still be categorised in "Primary outcome but not primary analysis".

2.3.14. If criteria (i.e. gender, age) are recorded as part of the randomisation process, these will be categorised under "randomisation items" even if they are also eligibility criteria.

2.4. What to do when data have been categorised

2.4.1. A single completed electronic data categorisation form should be returned to Shaun Treweek for collation (streweek@mac.com).

3. Associated documents

The list of data categories is at https://docs.google.com/spreadsheets/d/16jJq25M3pD79tjLEFBKwQxJjGCsX-0UKSJD2fE5bFk8/edit#gid=0

An example completed data categorisation form for the SALVO trial, together with trial protocol and data collection forms can be downloaded at https://app.box.com/s/6g6f6w1ltiozp982276509ssq6uv2q6s

4. Definitions

Data items: these are the individual pieces of data collected on the trial data collection forms. They are sometimes called fields or variables.
